# Determination of the Cancer Genome Atlas (TCGA) Endometrial Cancer Molecular Subtypes Using the Variant Interpretation and Clinical Decision Support Software MH Guide

**DOI:** 10.3390/cancers15072053

**Published:** 2023-03-30

**Authors:** Alexander Mustea, Damian J. Ralser, Eva Egger, Ulrike Ziehm, Sonia Vivas, Stephan Brock, David Jackson, Mateja Condic, Christian Meisel, Lucia Otten, Anna Laib, Miguel Cubas Cordova, Rahel Hartmann, Martin A. Stein, Dominique Koensgen, Matthias B. Stope

**Affiliations:** 1 Department of Gynecology and Gynecological Oncology, University Hospital Bonn, Venusberg-Campus 1, 53127 Bonn, Germany; 2 Molecular Health, Kurfuersten-Anlage 21, 69115 Heidelberg, Germany

**Keywords:** endometrial cancer, exome sequencing, molecular subtypes, TCGA

## Abstract

**Simple Summary:**

Besides the diagnosis of endometrial carcinoma (EC), the identification of EC subtypes is an important precondition for the effective treatment of the disease. Molecular factors have an important role in this context. Biomedical analysis is already very possible in experimental approaches. However, for clinical practice the procedures are often too complex, too expensive, and too time-consuming. In the present study, very good evaluated molecular markers were detected using an alternative method and compared with the original method. Analyses showed very good results. The important advantage of the new procedure is that the required molecular data can be obtained exclusively by sequencing. This may greatly simplify EC subtype classification and could be more easily incorporated into routine clinical diagnosis.

**Abstract:**

Background: The Cancer Genome Atlas (TCGA) network (United States National Cancer Institute) identified four molecular endometrial cancer (EC) subtypes using an extensive multi-method approach. The aim of this study was to determine the four TCGA EC molecular subtypes using a single-method whole-exome sequencing (WES)-based approach provided by MH Guide (Molecular Health, Heidelberg, Germany). Methods: WES and clinical data of n = 232 EC patients were obtained from TCGA. The four TCGA EC molecular subtypes designated as (i) Mutated Polymerase ε (POLE), (ii) Microsatellite Instability (MSI), (iii) Copy Number (CN) low and, (iv) CN-high were determined using the MH Guide software. The prognostic value of the subtypes determined by MH Guide were compared with the TCGA classification. Results: Analysis of WES data using the MH Guide software led to the precise identification of the four EC molecular subtypes analogous to the TCGA classification. Both approaches displayed high concordance in terms of prognostic significance. Conclusions: The multi-method-based TCGA EC molecular subtypes can reliably be reproduced by the single-method-based MH Guide approach. The easy-to-implement single-method MH Guide approach represents a promising diagnostic tool.

## 1. Introduction

Endometrial cancer (EC) is the sixth most common cancer in women worldwide, with more than 11,000 newly diagnosed cases and about 2700 cancer-related deaths in Germany per year [1,2]. Historically, EC is divided into two subgroups based on their histological characteristics: endometrioid (type I) and non-endometrioid (type II) EC [3,4,5,6]. Eighty percent of EC are type I tumors with an overall good prognosis and a 5-year survival rate of above 80% [7,8]. Type II EC, comprising primarily serous-papillary, clear-cell or undifferentiated histological subtypes, shows a more aggressive behavior accounting for a disproportionate number of EC-related deaths with regard to their frequency (40% of EC-related deaths, whereas they only account for 10 to 20% of all EC cases) [9]. Since EC type II has a significantly poorer prognosis, a more complex oncological therapy approach is required, including more extensive surgery with lymph node resection and subsequent chemo- and radiotherapy.

Several trials have shown that genomic profiling improves the risk assessment of patients with early-stage EC, thereby enabling individualized therapeutic approaches to optimize clinical outcomes [10,11]. The Cancer Genome Atlas (TCGA) network of the United States National Cancer Institute has previously reported an integrated multi-omics characterization (genomics, transcriptomics, proteomics, and methylomics) of n = 373 EC using a multiplatform strategy applying array- and sequencing-based technologies [12]. Based on this characterization, EC was classified into four distinct and prognostically significant subtypes: (i) Mutated polymerase ε (POLE) EC, a rare subtype that is characterized by a high number of single nucleotide variants, a specific mutation spectrum, and with excellent prognosis across all disease stages; (ii) Micro-satellite instability (MSI) EC is characterized by mismatch repair deficiency, high mutation stress, and moderate prognosis; (iii) Copy number (CN) low EC is an endometrioid subtype with intermediate good prognosis, including all remaining tumors that do not meet the criteria of the other EC subgroups; (iv) CN-high EC includes all serous EC cases of the study and is characterized by high levels of somatic copy number alterations and a poor prognosis. The findings of this retrospective molecular study suggest that EC is genetically heterogenous even within the same histological subgroup.

Moreover, the prognostic value of these four molecular EC subtypes with distinct clinical courses is of highest clinical relevance as it could be applied to therapy stratification. For instance, the POLE subtype was shown to have a significantly better progression-free survival (PFS) than the CN-high subtype, which was associated with the worst clinical outcome [12]. Characterization of patient-specific prognostic criteria based on the TCGA classification requires a complex methodological multi-omics approach that is resource intensive and difficult to implement in diagnostic routine procedures. Two different classification systems have been established in clinical routine, namely TransPORTEC and ProMisE, which are both based on molecular classifiers [13,14]. However, these classifications also require the use of two different methods: immunohistochemistry and Sanger sequencing/NGS.

In the present study, EC molecular subtypes according to the TCGA classification were reproduced using an innovative single-method analysis tool based on WES. Raw sequencing data from TCGA [12] were analyzed using the treatment decision support software MH Guide (Molecular Health, Heidelberg, Germany) with assignment of the molecular subtypes POLE, MSI, CN-low, and CN-high. Results were examined with regard to reproducibility of the TCGA classification data.

## 2. Materials and Methods

TCGA data processing: Raw sequencing data from n = 232 patients from the TCGA EC cohort were available from the Genomics Data Commons (GDC) for protected use under the project ‘Investigation of Cancer Subtyping Based on NGS Data’ (project ID 12045). Clinical data was downloaded from Firehose (age, race, ethnicity, weight, height, stage, grade) and cBioportal (PFS and overall survival (OS)), and supplemented with molecular subtypes (MSI, CN, integrative cluster). Aligned reads (bam format) from whole-exome DNA sequencing (Illumina GAIIx or HiSeq 2000 platforms) of primary tumor and matched blood (preferred) or tissue-derived normal samples were downloaded from the GDC legacy archive. Paired-end sequencing reads were extracted from alignment files using PicardTools (version 1.115) and SamToFastq (validation_stringency to lenient and include_non_pf_reads to true). Potentially existing unpaired reads were discarded.

MH Guide classification: Read files (fastq format) from raw sequencing data from n = 232 patients from the TCGA EC cohort were classified using the oncology treatment decision support software MH Guide (version 4.1.2; Molecular Health, Heidelberg, Germany). MH Guide provides a full bioinformatics pipeline plus clinical annotation of variants called single nucleotide variants (SNV), short indels, somatic CN alterations, and MSI status. POLE and MSI tumors were classified in the same manner as TCGA classification [12], using SNV and MSI status identified by MH Guide. To classify POLE ultramutated EC, somatic SNV that met the standard filtering criteria of the MH Guide for paired whole-exome analyses were used. Tumors with >500 SNV, a CA rate of >0.2, and a CG rate of <0.03 were classified as POLE. The remaining cases were classified as MSI if they were MSI high. After identification of POLE and MSI group members, remaining tumors were further classified as CN-high if they were predicted to fall into the CN 4 cluster.

To reproduce the CN-high class of TCGA classification, we trained a supervised classification model to predict the CN cluster 4 of TCGA from the WXS-based copy number calls of MH Guide. Cross-validation accuracy, precision, recall, and f1 were used to evaluate how well the classification based on DNA NGS data reproduced the original three platform molecular subtypes of TCGA. CN clusters were extracted from the original data set [12] and binarized into 1 if a case belonged to cluster 4 and 0 if a case belonged to cluster 1, cluster 2, or cluster 3. In total, 26% of samples fell into the CN cluster 4 class and 74% fell into the other classes. Multiple supervised classification algorithms and feature sets were tested with 5-fold cross-validation. The model was trained using 240 samples with available CN clusters, and classification performance was evaluated using stratified 5-fold cross validation. Models were implemented in Python 3.6.8 using package scikit-learn 0.22.1.

A naïve Bayes classifier with the following features was selected: Number of CN gains/CN losses per sample, number of CN gains/CN losses per chromosome, number of CN gains/CN losses per gene for the 25 most abundant genes, and ploidy and length of CN alterations per mega base. Although our features violate the assumption of uncorrelated features for a naïve Bayes classifier, this model showed robust performance in predicting CN cluster 4 with average cross-validation accuracy, precision, recall, f1-score, and ROC-AUC of 91.25%, 83.51%, 85.77%, 83.98%, and 95.08%, respectively, for the 240 training samples.

Statistics: The MH Guide classification results were evaluated using accuracy, precision, recall, and f1 scores with weighted averaging across sources to consider the imbalance of classes. The risk stratification performance of both classifications was assessed using Kaplan–Meier survival curves and multivariable log-rank tests. The chi-square test was used to test for a difference between class distributions of different classification methods. 

## 3. Results

The MH Guide algorithm was applied to the publicly available TCGA EC cohort comprising n = 232 patients (Table 1) [12]. According to the TCGA classification, 17 cases in this EC cohort were classified as POLE, 65 as MSI, 90 as CN-low and 60 as CN-high. For POLE, the MH Guide algorithm identified 100% (17/17) of POLE cases as determined by TCGA classification criteria. For the MSI subtype, 89% (58/65) of all MSI cases designated by TCGA classification were detected by the MH Guide algorithm. In total, 9% (6/65) of the undetected MSI cases were classified as CN-low and 2% (1/65) as CN-high. Analysis of the CN-low subtype revealed that 89% (80/90) were accurately identified as CN-low, but 11% (10/90) were found to be CN-high. The largest discrepancy between the two methods was observed in the identification of the EC subtype CN-high. Here, the MH Guide algorithm resulted in 85% (51/60) correctly identified CN-high EC. The undetected 15% (9/60) of cases were classified as CN-low.

**Figure 1:**
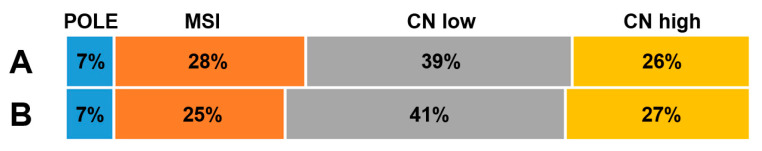
Distribution of EC subtypes POLE, MSI, CN-low, and CN-high in the EC cohort (n = 232) after application of TCGA (**A**) and MH Guide algorithm (**B**).

Application of the MH Guide algorithm to the TCGA EC cohort led to consistency with respect to identification of molecular EC subtypes in approximately 89% of the cases. There were slightly differing results in a few individual cases. In particular, among the EC cases with unfavorable prognosis, individual patients were assigned to different subtypes according to both classification systems, as indicated in Table 1. However, the overall comparison of TCGA and MH Guide classification showed comparable results (Figure 1). The distributions of POLE (TCGA: 7%; MH Guide: 7%), MSI (TCGA: 28%; MH Guide: 25%), CN-low (TCGA: 39%; MH Guide: 41%) and CN-high (TCGA: 26%; MH Guide: 27%) were identified in very similar proportions in the TCGA EC cohort by both methods (chi-square test, *p* = 0.9041).

Identification of the four molecular EC subtypes based on whole-exome sequencing data using the MH Guide algorithm resulted in good overall cross-validation accuracy, precision, recall, and f1-score of 88.78%, 91.92%, 90.76%, and 91.01%, respectively when using macro-averaging, and 88.78%, 89.61%, 88.78%, and 88.82%, respectively when using weighted averaging.

**Figure 2:**
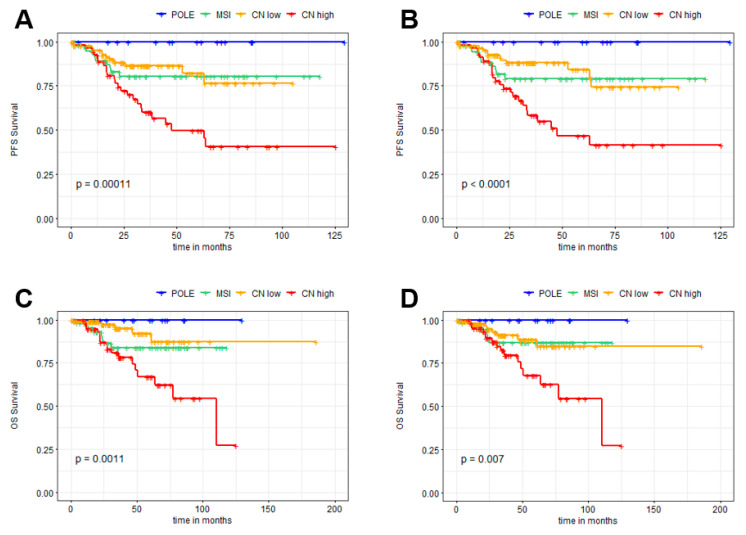
Kaplan–Meier survival curves for progression-free survival (PFS; (**A**,**B**)) and overall survival (OS; (**C**,**D**)) of the EC cohort according to TCGA classification (**A**,**C**) and MH Guide algorithm (**B**,**D**). Due to lack of clinical data, the number of patients in the respective groups differed (PFS: n = 220, OS: n = 232). Log-rank p-values are given as inserts.

To further evaluate the distributions of the different EC subtypes according to both methods, survival analyses were performed. In line with the results presented above, the Kaplan–Meier survival analyses demonstrated a very comparable overall pattern (Figure 2).

Both PFS and OS demonstrated comparable values for POLE, MSI, CN-low, and CN-high subtypes. The 36-month PFS rates (n = 220, Table 2) for the original TCGA molecular subtypes were 1.00, 0.81, 0.87, and 0.60 for the POLE, MSI, CN-low, and CN-high classes, respectively; for the MH Guide algorithm based molecular subtypes, PFS rates were similar with 1.0, 0.79, 0.88, and 0.58 proportions for the POLE, MSI, CN-low, and CN-high classes, respectively. The 36-month OS rates (n = 232, Table 3) according to TCGA were 1.00, 0.84, 0.95, and 0.81 for POLE, MSI, CN-low, and CN-high, respectively; with consistent OS rates obtained for EC subtypes according to the MH Guide algorithm: 1.00, 0.87, 0.91, and 0.82 for POLE, MSI, CN-low, and CN-high, respectively. Overall, these differences were not statistically significant.

## 4. Discussion

According to the WHO classification, seven histopathological EC types are differentiated: endometrioid, serous, clear cell, mixed, neuroendocrine, undifferentiated and dedifferentiated carcinomas, carcinosarcoma, and unusual carcinoma types [15]. These histologic subtypes are further summarized into type I and type II carcinomas. Pathological diagnosis remains a key tool for stratifying EC subtypes. Histopathological subtyping is often interobserver dependent with high variation rates, especially for high-grade EC types due to similar or identical immunohistochemical appearance [16]. Clinically, accurate EC subtyping is crucial for determination of the individualized therapy approach for each patient. In this context, classification by molecular markers can lead to higher confidence and prognostic power [17]. Therefore, in addition to conventional histopathology, a specific panel of markers is required to obtain further diagnostic/prognostic information in individual cases.

Molecular diagnostics are increasingly important in modern, individualized oncology as they enable therapy stratification to improve PFS and OS on the one side and to reduce therapy-related toxicity and to increase safety on the other side. The four TCGA EC molecular subtypes, namely POLE, MSI, CN-low, and CN-high, enable further stratification of EC patients allowing individualized treatment decisions. Patients with POLE hypermutated EC, typically displaying high-grade endometroid carcinomas, exhibit an excellent prognosis. In this setting, overtreatment by means of adjuvant chemotherapy can be avoided. In contrast, the CN-high subgroup, which comprises EC with serous-like histology and p53 gene mutations, exhibits a very poor prognosis and requires an extended therapy approach. Hence, it is critically important to identify the different EC subtypes in order to avoid under-treatment of high-risk patients and over-treatment of low-risk patients.

With regard to prognosis, the EC subtypes MSI and CN-low are considered intermediate. The MSI subtype is characterized by high mutation rates, hypermethylation, and a frequently hypermethylated MLH1 promoter. Histopathologically, CN-low EC are endometrioid carcinomas with low copy number alterations including EC with nonspecific molecular profiles. For both subtypes, MSI and CN-low, prognosis is considered intermediate.

For MSI patients, there is an increased risk of developing further cancer (e.g., Lynch syndrome) [18,19]. In this context, it is strongly recommended that patients undergo human genetic counseling and are enrolled in appropriate screening programs. In terms of oncological therapy, administration of checkpoint inhibitors has recently been approved in the United States and Europe. Thus, in addition to the prognostic significance, a therapeutic relevance also results from the knowledge of the MSI status. It can be assumed that further immune checkpoint inhibitors will be approved for the therapy of specific EC subtypes, which means that the specific and sensitive detection of MSI criteria may play an even greater role in therapy decisions in the future.

In the recently published PORTEC3 study, a multicenter, randomized, prospective phase III trial, molecular EC subtyping comparable to the TCGA classification was performed with regard to clinical outcomes. This study confirmed the strong prognostic value of molecular EC subtypes regardless of the histologic type. Especially, patients with POLE hypermutated tumors had excellent clinical outcomes [14,20]. These findings are incorporated in the ongoing PORTEC4 trial with therapy stratification based on molecular subtyping [21,22].

As a consequence, EC subtyping is recommended in both the European EC guidelines and the EC guidelines of the National Comprehensive Cancer Network in the USA [23].

The molecular EC subtypes defined by TCGA in 2013 mark a milestone in the understanding of this gynecologic malignancy and represent a step towards individualized therapy in EC patients. However, this classification requires an extensive multi-omics approach, making it impractical in routine clinical practice. Due to this not inconsiderable effort, the original molecular subtypes have not yet been evaluated in prospective trials. Molecular subtyping of EC, i.e., prognostic evaluation of the methodology and its introduction into routine diagnostics, would benefit considerably if it could be performed with a single routine laboratory method. In current clinical routine, molecular EC subtypes are determined by molecular surrogate markers analogous to the TransPORTEC or ProMisE, algorithm [13,14]. Both algorithms are based on a two-methods approach, namely immuonhistochemistry and sequencing (Sanger or NGS). In the present study, we established the MH Guide algorithm that was able to reproduce the molecular subtypes according to TCGA in the original TCGA EC cohort by whole-exome sequencing. Comparison of the molecular subtypes according to the MH Guide algorithm with the original TCGA subtypes revealed no significant differences in the differentiated molecular subtypes. The subtle discrepancies in subtype determination (Table 1) are attributable to differences in methodology. Further, the prognostic value of the distinct molecular subtypes determined using the MH Guide algorithm is equal compared to the TCGA methodology. This is of highest relevance from a clinical point of view as slightly differing molecular subtype determination is not impacting clinical decisions. Hence, the described MH Guide algorithm allows a classification procedure based on only one method instead of the original TCGA multi-omics approach.

The implementation of a simple single method approach would not only facilitate evaluation of large international EC cohorts, but it is also the more feasible procedure in clinical routines at EC therapy centers. In addition, the sequencing approach of the MH Guide classification has the advantage of determining the mutation status of further possible target genes in terms of individualized therapy as BRCA-1/2, CTNNB1, or PTEN. This could further improve molecular subtyping and, since no additional laboratory procedures need to be installed, simplify molecular diagnostics in EC. Of note, identification of possible druggable mutations might delineate targeted treatment determination in later therapy lines of EC. This is a clear advantage over the currently used classification systems TransPORTEC and ProMisE.

## 5. Conclusions

The four molecular subtypes of EC according to TCGA classification are determined using a multi-method approach. The present study shows that this can also be performed based on WES data using MH Guide software. All four molecular TCGA EC subtypes (i) POLE, (ii) MSI, (iii) CN low, and (iv) CN high could be accurately identified after MH Guide analysis. The prognostic deductions from MH Guide analysis were also highly consistent with the original TCGA classification. The multi-method molecular subtypes of the TCGA classification system can be reliably reproduced by the single-method MH Guide approach. Thus, the easy-to-implement MH Guide approach represents a promising diagnostic approach for clinical use.

## Figures and Tables

**Table 1:** Comparison of EC molecular subtype designation based on TCGA [12] and MH Guide. Cases according to TCGA classification were reclassified using the MH Guide algorithm and indicated as patients in the respective EC subtypes. In parentheses, the percentage of subclassified patients is given relative to the total number in the subclasses as defined based on TCGA.

TCGA Classification		MH Guide Classification			
		POLE	MSI	CN-Low	CN-High
**POLE**	17/232	17 (100%)	0	0	0
MSI	65/232	0	58 (89%)	6 (9%)	1 (2%)
CN-low	90/232	0	0	80 (89%)	10 (11%)
CN-high	60/232	0	0	9 (15%)	51 (85%)

**Table 2:** The 36-month PFS of subtypes by original TCGA classification and based on the MH Guide algorithm.

	Original TCGA Classification 36-Month PFS (n)	MH Guide Classification 36-Month PFS (n)
POLE	1.00 (17)	1.00 (17)
MSI	0.81 (61)	0.79 (56)
CN-low	0.87 (88)	0.88 (91)
CN-high	0.60 (54)	0.58 (56)

**Table 3:** The 36-month OS of subtypes by original TCGA classification and MH Guide algorithm.

	Original TCGA Classification 36-Month OS (n)	MH Guide Classification 36-Month OS (n)
POLE	1.00 (17)	1.00 (17)
MSI	0.84 (65)	0.87 (58)
CN-low	0.95 (90)	0.91 (95)
CN-high	0.81 (60)	0.82 (62)

## Data Availability

The underlying raw data are made publicly available by The Cancer Genome Atlas (TCGA) network of the United States National Cancer Institute. The datasets generated and analysed during the current study are the property of Molecular Health and are not publicly available. They can be asked for from the corresponding author upon reasonable request.
